# Supplementary material for: Urinary Inflammatory and Oxidative Stress Biomarkers as Indicators for the Clinical Management of Benign Prostatic Hyperplasia
Source: Int J Mol Sci. 2025 Jul 6;26(13):6516. doi: 10.3390/ijms26136516 (PMC12249731; doi:10.3390/ijms26136516)
Supplement: Supplementary file 1 [file ijms-26-06516-s001.zip › ijms-3682633-supplementary captions.pdf]

Supplementary Figure S1. Scatter plots showing the correlations of the clinical characteristics and the baseline urinary biomarker levels in the medical group of clinical BPH patients. Correlations between VE and IL-1β (A), VE and IL-6 (B), VE and IL-8 (C), VE and TNF-α (D), and BWT and TAC (E).

VE, voiding efficiency; TNF-α, tumor necrosis factor-α; BWT, bladder wall thickness; TAC, total antioxidant capacity

Supplementary Figure S2. Scatter plots showing the correlation of the changes of clinical characteristics and the changes of urinary biomarker levels in the medical group of clinical BPH patients. Correlations between ΔIPSS-V and ΔIL-1β (A), ΔIPSS-V and ΔIL-6 (B), ΔIPSS-V and ΔIL-8(C), ΔIPSS-V and ΔTNF-α (D), ΔVE and ΔIL-1β (E), ΔVE and ΔIL-6 (F), ΔVE and ΔIL-8 (G), ΔVE and ΔTNF-α (H), ΔcQmax and ΔIL-1β (I), and ΔcQmax and ΔIL-8 (J).

Δ, change in [specific variable]; IPSS-V, International Prostate Symptom Score voiding subscore; VE, voiding efficiency; cQmax, corrected maximal urinary flow rate; TNF-α, tumor necrosis factor-α.

Supplementary Figure S3. Scatter plots showing the correlations between clinical characteristics and urinary biomarker levels in the surgical group of clinical BPH patients.

(A–C) Baseline correlations: (A) cQmax vs 8-isoprostane; (B) BWT vs IL-6; (C) BWT vs TAC.

(D) Post-treatment changes: ΔVE vs Δ8-OHdG.

cQmax, corrected maximal urinary flow rate; BWT, bladder wall thickness; TAC, total antioxidant capacity; Δ, change in [specific variable]; VE, voiding efficiency; 8-OHdG, 8-hydroxy-2-deoxyguanosine

Supplementary Figure S4. The enrollment flowchart illustrates the selection of clinical BPH patients and control participants, as well as the timing and components of clinical assessments and urinary biomarker measurements.
