# Supplementary figures and images for: Urinary Inflammatory and Oxidative Stress Biomarkers as Indicators for the Clinical Management of Benign Prostatic Hyperplasia
Source: Int J Mol Sci. 2025 Jul 6;26(13):6516. doi: 10.3390/ijms26136516 (PMC12249731; doi:10.3390/ijms26136516)

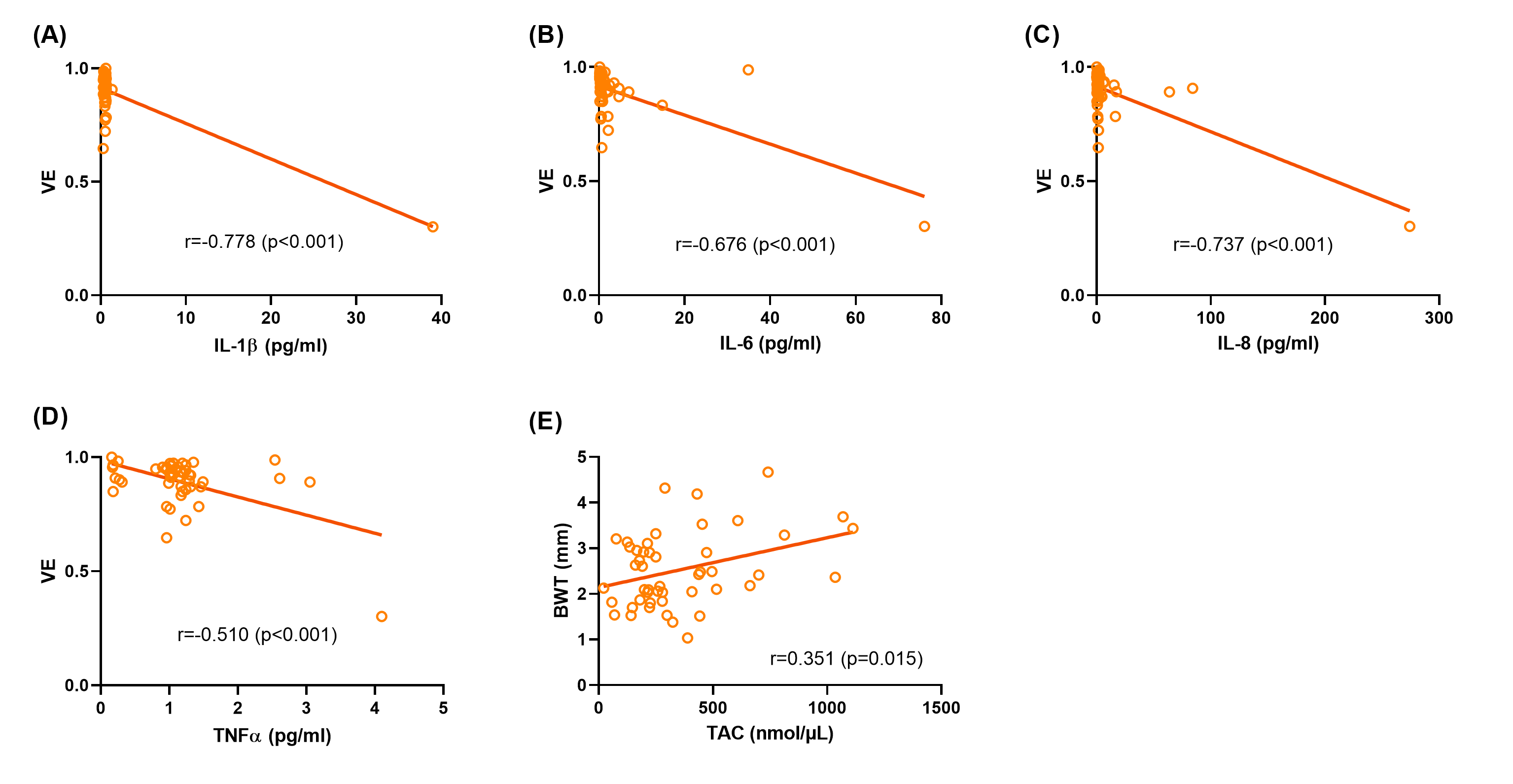

Supplement: Supplementary file 1 [file ijms-26-06516-s001.zip › Supplementary Figure S1 medical BPH_baseline.tiff]

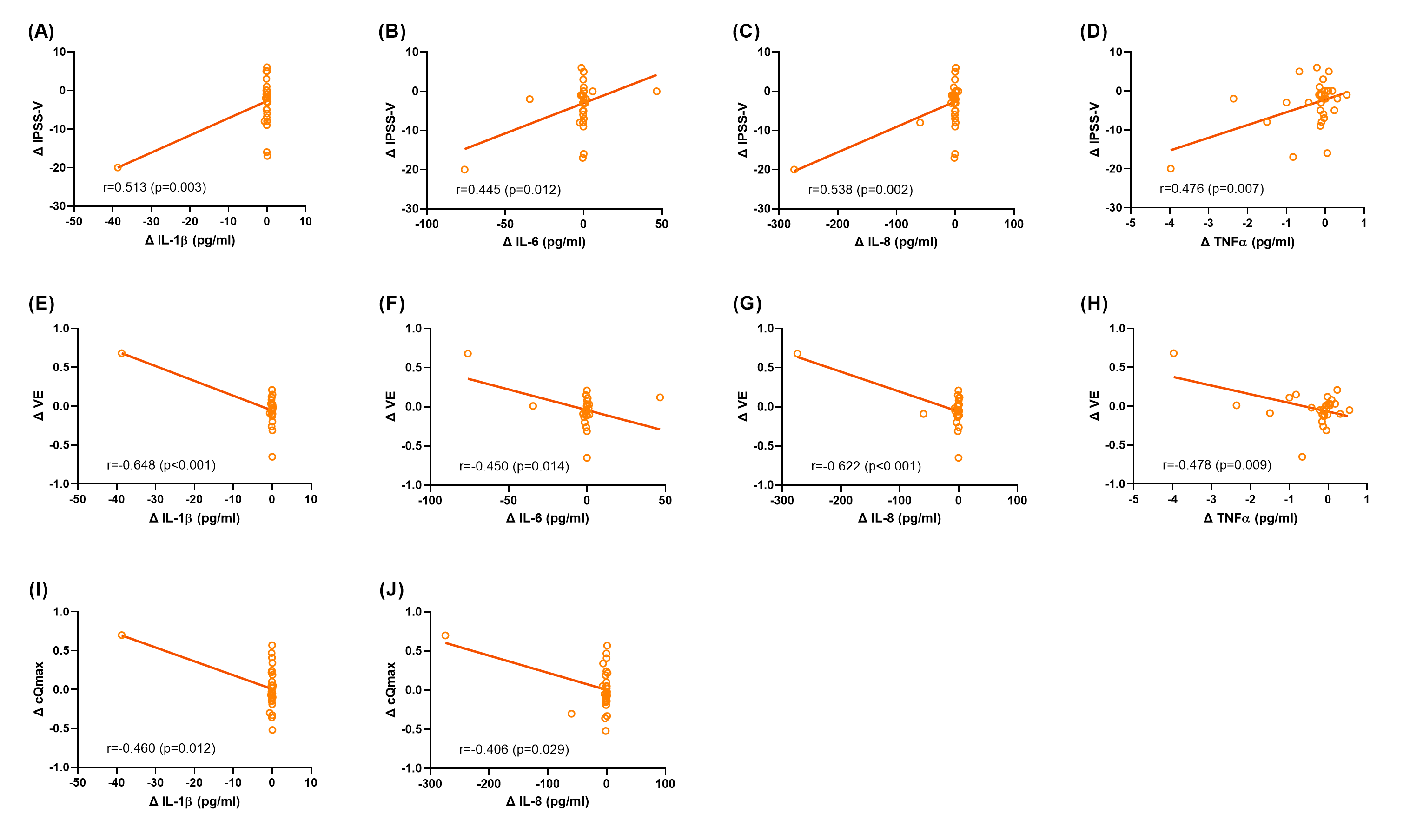

Supplement: Supplementary file 1 [file ijms-26-06516-s001.zip › Supplementary Figure S2 medical BPH_changes.tiff]

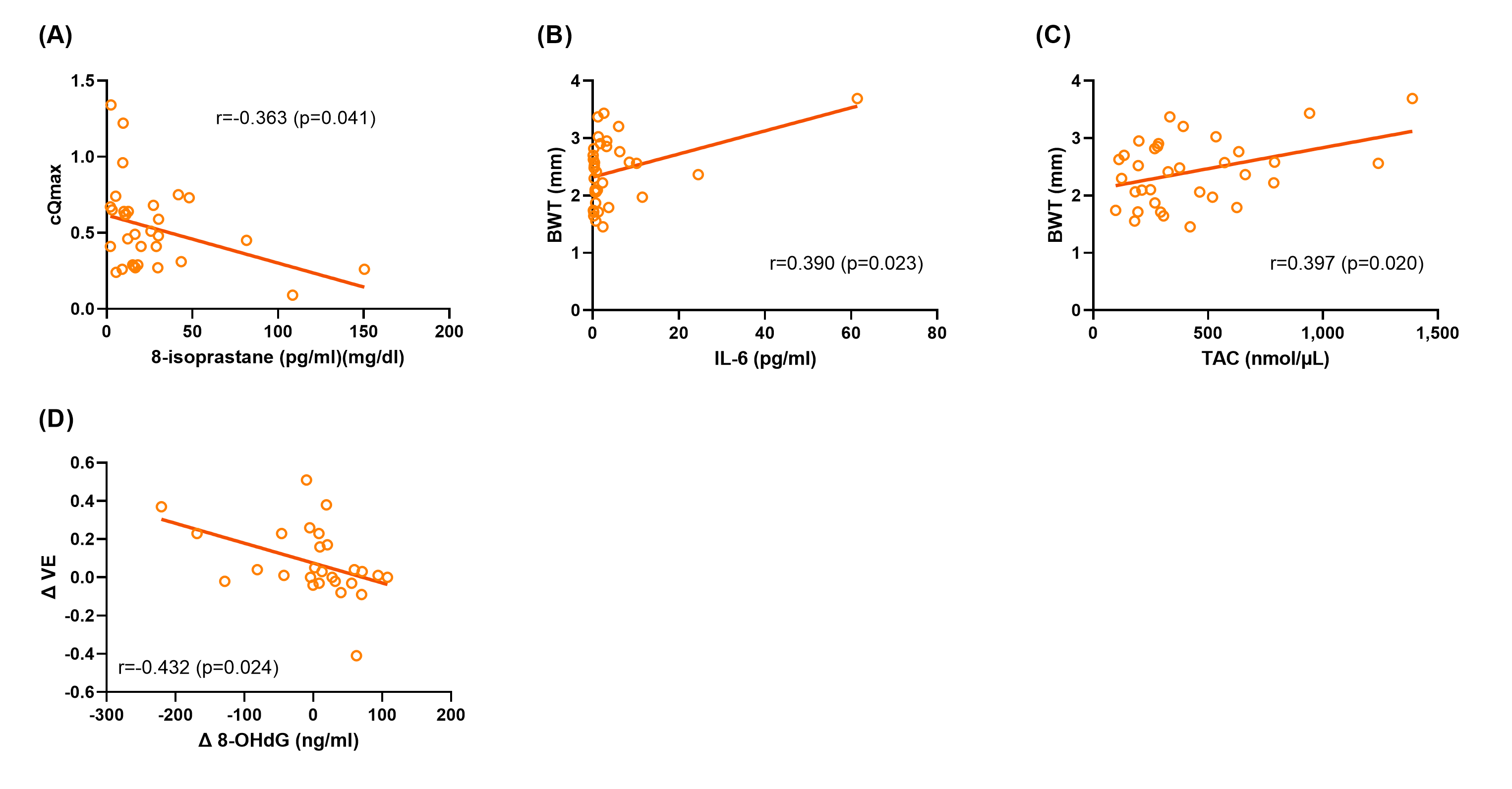

Supplement: Supplementary file 1 [file ijms-26-06516-s001.zip › Supplementary Figure S3 Surgical BPH.tiff]

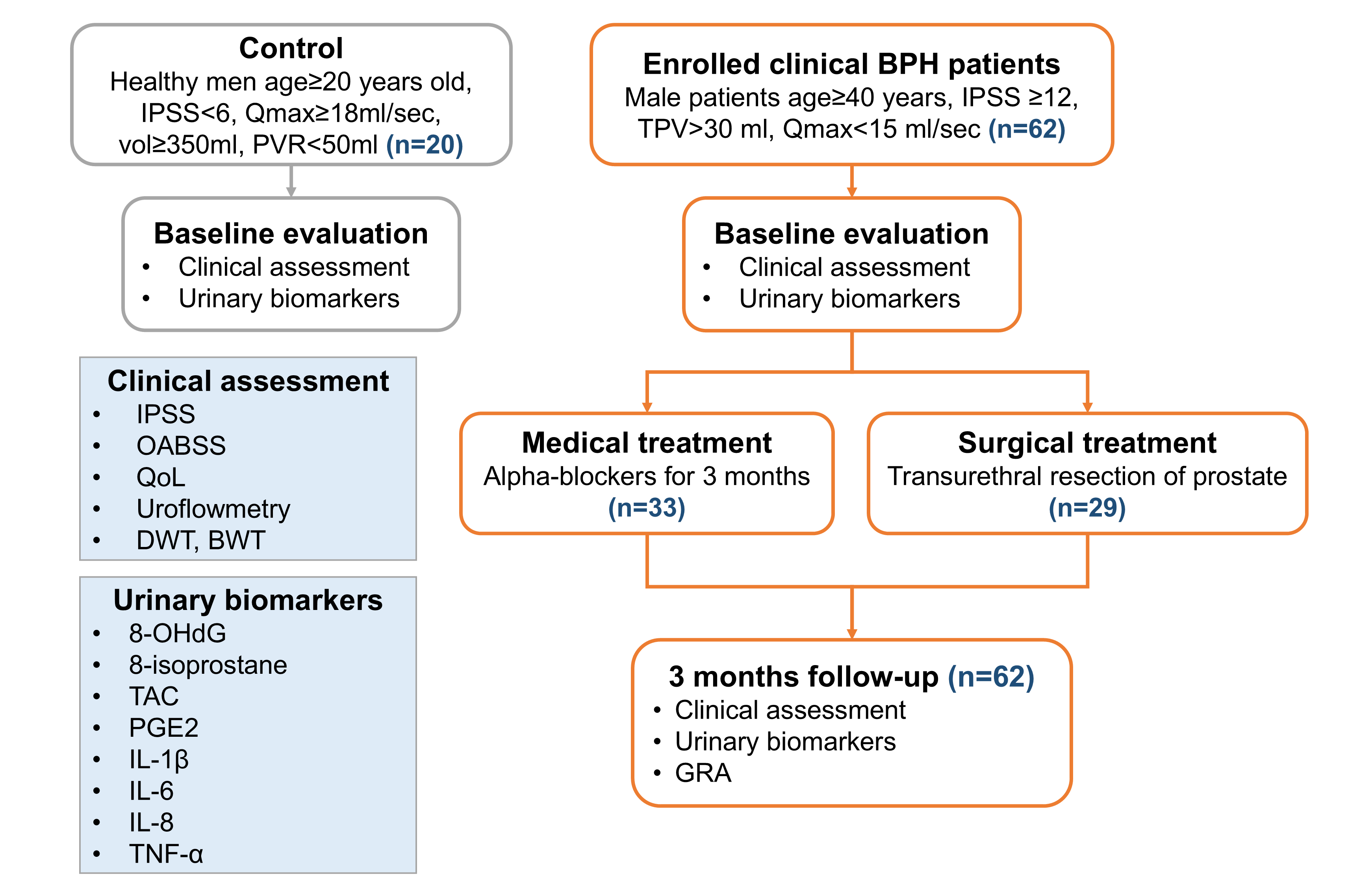

Supplement: Supplementary file 1 [file ijms-26-06516-s001.zip › Supplementary Figure S4_flow chart.tif]
